# Tracing the Single and Combined Contributions of Home-Grown Supply and Health Literacy on Fruit and Vegetable Consumption: An Empirical Exploration in Rural India

**DOI:** 10.3389/fpubh.2021.591439

**Published:** 2021-05-21

**Authors:** Yun-Hsuan Wu, Spencer Moore, Cameron McRae, Laurette Dubé

**Affiliations:** ^1^Department of Public Health, China Medical University, Taichung, Taiwan; ^2^McGill Centre for the Convergence of Health and Economics, McGill University, Montreal, QC, Canada; ^3^Health and Society Group, Wageningen University and Research, Wageningen, Netherlands; ^4^Desautels Faculty of Management, McGill University, Montreal, QC, Canada

**Keywords:** health literacy, agriculture, nutrition, fruits and vegetables, home-grown consumption

## Abstract

Low fruit and vegetable consumption (FVC) remains a global health challenge. Fostering subsistence agriculture through the production and home-grown consumption (HGC) of fruits and vegetables are seen as potential strategies for improving overall FVC, in particular, for developing countries like India. In addition, educational strategies targeting FVC health literacy are also used. Little evidence has documented a connection between these two strategies. We examine the single and combined influence of HGC and health literacy with regard to benefits from fruits and vegetable consumption. Data were collected from 427 rural households in the state of Odisha, India. Three outcomes were examined: FVC, as well as fruit and vegetables separately. Linear and Poisson regression were used to examine the association among home-grown consumption (HGC), FVC health literacy, and the FVC outcomes. Findings show that HGC, but not FVC health literacy, was directly associated with FVC (β = 0.65, SE = 0.10, *p* = 0.008) and vegetable consumption (β = 0.57, SE = 0.11, p = 0.02). However, both HGC (β = 0.58, SE = 0.05, *p* < 0.01) and FVC health literacy (β = −0.07, SE = 0.02, *p* = 0.001) were associated with fruit consumption. In addition, HGC effect is concentrated among participants who reported low FVC health literacy, especially on overall FVC and vegetables alone. Results are discussed in relation to the beneficial role played by HGC in those particularly vulnerable households who perceived little FVC health literacy. Our results provide insights on novel improved FVC consumption across all population segments. Future research should explore the complex interplay between agricultural policies and educational programs in the design of interventions promoting fruit and vegetable production and consumption.

## Introduction

Fruits and vegetables (FVs) are important components of a healthy diet, and when consumed in sufficient levels, it can reduce the risk of chronic diseases, such as diabetes and obesity, cardiovascular diseases, and certain types of cancer (1–5). The Food and Agriculture Organization of the United Nations/World Health Organization (FAO/WHO) recommends a daily individual intake of at least 400 g of FVs a day, which is the equivalent of five servings of 80 g each (6). Yet, in spite of robust evidence for the benefits associated with normative fruit and vegetable consumption (FVC), the majority of people consistently consume less than the daily recommended FV intakes (6–9). In 2017 alone, it is estimated that inadequate fruit and vegetable consumption led to an estimated 3.9 million deaths worldwide (10).

Among people living in low- and middle-income countries (LMICs), 78% consume less than the daily recommended FVC (7, 8). Low FVC has been recognized as a leading dietary risk for LMICs (11), and therefore, its contribution to the global burden of diseases and food insecurity are particularly pressing (12, 13). Recently, there has been an increased strategic focus on multisectoral convergence for nutrition-sensitive programs—programs that involve diverse sectors, such as agriculture, education, health, water, and sanitation—in order to affect the underlying determinants of nutritional issues (14–18). One of the key nutrition-sensitive pathways through which agriculture can impact nutrition in developing countries, like India, is food access from home-grown produce (14, 19). Home-grown produce may include at-home gardens for average consumers in urban areas (20) or, for farmers in rural areas, only one element of a broader homestead food production system. Agricultural produce from homesteads cannot only be sold to earn income for food expenses but also can be used for people's own household consumption (19, 21, 22).

Direct linkages between agricultural production and nutrition are created by household decisions regarding the quantity and composition of food production, which are partially reliant on the needs of the household (23). Surendran et al. recently documented home-grown consumption as a key pathway through which peri-urban households in Hyderabad, Telangana (approximately 1,000 km southwest of our study site), sourced food for consumption, with support tied strongest to vegetables that are cultivated more ubiquitously, when compared with fruits, among the farmers they interviewed (24). Studies in developed countries have also supported this farm-level pathway to nutrition, with empirical evidence for adults residing in the United Kingdom (25), as well as children living in the rural United States (26). Despite the intuitive appeal of home-grown consumption (HGC) as means of increasing FVC, there is scant empirical evidence to support these initiatives and a need for more, and better, research to support program design (27, 28). This is in part due to the disconnection between agriculture, nutrition, public health, and economics research, combined with the lack of survey data that links these various fields (23). For instance, information about agricultural production is often missed in nutrition surveys, whereas anthropometric and nutritional measures may be lacking in agricultural and economic surveys (19). Again, in India, a recent exploratory analysis of factors underlying inadequate and unequal fruit and vegetable consumption using National Sample Survey Office (NSSO) data did not measure agricultural production or home-grown consumption within the same study and, therefore, did not identify it as a contributing factor, which again highlights the disconnect among sectors (29).

Beside the agriculture sector, education and knowledge-based interventions targeting behavior change are often used as a means to improve FVC by increasing health literacy, such as nutrition knowledge, nutritional benefits, dietary guidelines, core food group intake, or specific nutrition education to prevent or manage diseases (30). For instance, perceived benefits of eating FVs are associated with actual FVC (31, 32). Several components of health literacy are tied to FVC, including disease prevention, health promotion, and general health literacy (33). Maternal health literacy is also associated with early childhood development, where high health literacy has been observed to reduce the likelihood of childhood stunting and underweight among children in rural and urban India (34). Other studies have also documented the association between health literacy and FVC in high-income economies (35–38). Yet, few studies have assessed the relative contribution of agriculture strategies and nutrient health literacy to explain FVC in low-income settings, which is particularly important to know for developing countries and emerging economies where households perceive more barriers toward changing FVC behaviors (9, 39). While a recent review of nutrition-sensitive programs led by Ruel et al. suggests that agriculture programs could be more effective at improving maternal and child nutrition outcomes when incorporated with nutrition and health behavior change communications, little empirical evidence exists to support this linkage (40).

India is the second largest agricultural producer of FVs in the world, accounting for roughly 10 and 15%, respectively, of total global production (7). Traditionally, the Indian diet had a strong affinity for FVs. Yet, surveys in India have indicated low levels of FVC (41, 42), and a prevalence of low FVC as high as 74% among adults (8). The most recent research indicates that, according to the nationwide NSSO Household Consumer Expenditure Survey 2011–2012, the per capita household intake are 145 g for fruits and 15 g for vegetables per day for rural India, and 155 and 29 g, respectively, for urban India (43). Both rural and urban FVC fall lower than that of the recommended intake value. According to a recent study in rural India (44), home-grown consumption can mediate the influence of farm-level interventions targeted at increasing consumption of FVs. However, FVC is the product of a complex interaction of factors, and only limited studies have assessed the independent and interacting influences of agricultural- and knowledge-based drivers on improving FVC, especially in India.

Additionally, the drivers of eating behaviors, including food choices and consumption, are the results of the complex interplay among sociodemographic, psychosocial, and environmental factors (45). Therefore, a single-faceted contribution of knowledge or agriculture might be not enough to improve FVC at the recommended levels that it requires (46). A limited number of studies have examined drivers of FVC specifically despite much work being done for food overall, including the impact of availability, accessibility, affordability, appeal, accommodation, and acceptability of consumption [for review, see Caspi et al. (47); French et al. (48); Glanz et al. (49); Dubé et al. (50); Dubé et al. (51); Chandon and Wansink (52)]. As rural areas open to modern agri-food development and urbanization, the expansion of retail marketing is expected to provide a steady, affordable supply of FVs, but evidence also remains limited (53, 54).

Although some empirical work from rural India has shown that home-grown fruit and vegetable consumption (HgFVC) can mediate FVC (44), it is not clear whether and how FVC health literacy could modify the association between HgFVC and FVC. Therefore, this study addresses this gap by not only evaluating the relative contributions of HgFVC and health literacy in rural India but also examining the modifying role of health literacy. In addition, this study further estimates whether the influence of HGC and other drivers on FVC may vary by different levels of health literacy.

## Methods

### Data

Data were obtained from the baseline survey of an agri-food value chain intervention sponsored by Grand Challenges India (44). The intervention was implemented by eKutir, a social enterprise in the state of Odisha, India, that leverages both information and communication technology (ICT) platform-enabled ecosystem and a micro-entrepreneurial deployment strategy to solve food and nutrition insecurity in low-resource rural communities. eKutir provides inputs, technical assistance, market linkages, and daily market pricing information through ICT tools deployed throughout their ecosystem. The evolution of eKutir's ICT-enabled ecosystem (55) as well as its impact on FVC for rural farmers and urban consumers (44) have previously been studied.

The baseline survey for this study was conducted in four districts of rural Odisha: Kandhamal (I and II), Jharsuguda, Angul, and Nayagarh. The surveys were administered between April and May 2015. Study sites were selected based on existing geographical boundaries, eKutir operational requirements, and consumers' and farmers' voluntary willingness to participate in the evaluation of the program. The following criteria were used to identify rural village sites: (i) high farmer density, (ii) procurement logistics, and (iii) the capacity for hosting a VeggieLite center. Within villages, the number of households selected was proportionate to the village population size. A total of 32 villages with 427 households were selected from the four districts for baseline data collection. Structured questionnaires were administered to participating rural households. The questionnaire included specific modules on (1) household demographic information, (2) cropping pattern and agriculture inputs, (3) fruit and vegetable consumption patterns, and (4) household development and poverty index. Ethical approval was obtained from the Research Ethics Board of McGill University. Informed consent was also collected from all participants.

## Measures

### Main Outcomes

FVC was based on self-reported information from each rural household. To calculate the overall consumption for FVs, rural households were asked to name the top five vegetables and fruits that they had consumed in the past 7 days, as well as the quantity, source, and amount they purchased vs. what they grew at home. As per India's dietary guidelines, potatoes were included as vegetables in the calculations. The amount of five vegetables consumed per household in the past 7 days was summed to calculate the total grams of vegetables consumed per household. The total grams of vegetables consumed per household was then divided by the household size and 7 days, in order to calculate the average grams of vegetables consumed per person per day. To arrive at the average daily servings of vegetables per person, the average grams of vegetables consumed per person per day was divided by 80 (56). The average daily servings of fruits per person were calculated in the same manner. Overall, daily servings of FVs were the sum of vegetable and fruit servings.

### Independent Variables

To capture a driver from the agricultural sector, we measured households' level of home-grown consumption (HGC) following four steps: (1) For each reported vegetable, we calculated the amount that households consumed in total grams as a function of the amount that the household consumed in the past 7 days multiplied by the percentage reported to have come from home-grown production, (2) we divided the household HGC total by the household size, (3) we divided this total by 80 to get the average daily servings of vegetable per person, and (4) we summed the amount of daily servings for the top five vegetables (or fruits) consumed from home-grown production.

As for measuring the household health literacy, we asked the participants about their beliefs regarding FVs as a proxy. Using a five-point Likert scale from not very important to very important, participants responded to six items asking, “how important they believe vegetables were for.” The six items were (1) improving your family health, (2) good health and nutrition, (3) your body, (4) your eyes and bones, (5) providing nutrients that your body needs, and (6) enhancing immunity/body fighting against disease. Due to the medium to high correlation between these items (Pearson correlation coefficient range: 0.39–0.61), a composite health literacy score was constructed by the weighted sum of six items. The weights were the standardized scoring coefficients from the first component of a principal component analysis due to 58% of the variability accounting for the first component. High internal reliability justified the use of the composite score (Cronbach's alpha = 0.86). In order to further explore the differential influence of homegrown consumption by the extent of health literacy, we stratified our sample into two groups based on the median value of the composite fruit and vegetable health literacy score for subpopulation analyses.

To measure household demand drivers, participants were asked how the following factors affected their decision to purchase FVs: (1) affordability, (2) availability, (3) accessibility, (4) proximity (5) taste (6) easiness to prepare/cook (7) knowledge to prepare FVs, and (8) food safety of FVs. Affordability means people can afford to purchase FVs. Availability is defined as the ability to purchase FVs that the household prefers to eat. Accessibility represents the ability to go to a place where fruits and vegetables are sold. Proximity indicates the nearness of store/place where FVs can be purchased. Indicator responses were on a five-point scale to represent the extent of agreement (1 = strongly disagree, 2 = disagree, 3 = somewhat agree, 4 = agree, and 5 = strongly agree).

### Control Variables

Household socio-economic and -demographic characteristics were included as control variables, including area of irrigated land, education level, primary language spoken, social groups, and household food insecurity. The highest level of education completed by the head of household and the area of irrigated land were included as proximal measures for socio-economic status (SES) for this analysis due to the low reliability of self-reported income collected for this study (44). The primary language was classified into one of the four categories: (1) Odia, (2) Sambalpur, (3) Adivashi, and (4) Other. Social group is one of the greatest determinants of health outcomes in India (57), and for this study, social groups were categorized into four groups: (1) general, (2) scheduled caste (SC), (3) scheduled tribe (ST), or (4) other backward caste (OBC). Among the caste systems, scheduled tribes and scheduled castes are the most socially disadvantaged groups in India (57). Household food insecurity was measured using two questions: (1) “During the last year, was there ever no food to eat of any kind in your house because of lack of resources to get food?” and (2) “Is anybody in your household severely undernourished?” If participants responded no to both questions, they were defined as food secure. However, if they answered yes to either one or both, they were defined as food insecure.

### Statistical Analyses

In this study, households were removed if they were missing information on fruit and vegetable consumption, as well as health literacy, yielding a final analytical sample of 421 households. Descriptive analyses for continuous variables were reported with means and standard errors, and categorical variables were reported with percentages for the whole sample. Additional comparisons between low- and high-health literacy groups were assessed using the *t*-test for continuous variables with normal distribution as well as the Wilcoxon Mann–Whitney *U*-test for continuous variables without normal distribution and chi-square test for categorical variables. Using overall FVC as the main outcome, a multivariable regression model was then built to evaluate the association of home-grown fruit and vegetable consumption (HgFVC) and other drivers with FVC after adjusting all control variables, including eight household demand indicators, area of irrigated land, education, primary language spoken, social group, household food insecurity (Model 1). In Model 2, the interaction term between HgFVC and health literacy was added to Model 1 to examine whether the influence of HgFVC on FVC may vary with health literacy. Following the same modeling process, analyses were conducted separately for average daily servings of (1) vegetables per person and (2) fruits per person. Since the distribution of average daily servings of fruits and vegetables per person, as well as vegetables per person, were close to normal, linear regressions were used for the modeling process. Poisson regression was used for the outcome of average daily servings of fruits per person due to its skewed distribution toward zero servings. Since the interactions between HGC and health literacy for the three outcomes were all significant, subpopulation analyses were conducted to further assess if the influence of HGC and other drivers might differ between the low- and high-health literacy groups. An *a priori* power calculation shows that based on 15 predictors, and a two-sided significance level of 0.05, 139 participants would provide 80% power to detect a medium effect size f^2^ of 0.15 by using G^*^Power 3.1.9.7 software (58). All analyses were conducted in STATA, version 14 (Stata, College Station, TX, USA).

## Results

Descriptive analyses are shown in Table 1. The mean value of daily FV servings per person was 3.35 (SD = 1.69), with average daily vegetable servings of 3.13 (SD = 1.57) per person and average daily fruit servings of 0.22 (SD = 0.52) per person. Among the participants, 87% did not reach the recommended levels of five or more servings of fruits and vegetables per day. Specifically, 54% of participants did not consume three or more servings of vegetables daily per person, only 1% of participants (*n* = 5) consumed two or more servings of fruits daily per person, and around 69% of participants consumed zero servings of fruits daily. When comparing between the low- and high-health literacy groups, there were no differences for overall FVC and vegetable consumption (VC) alone, but the high-health literacy group had significantly lower fruit consumption (FC) compared with the lower one.

**Table 1:** Descriptive information for all, low-, and high-health literacy groups.

	**All**	**Low**	**High**	
	**Mean (SD)**	**Mean (SD)**	**Mean (SD)**	*p*-value^*^
Daily servings per capita				
Fruits and vegetables	3.35 (1.69)	3.29 (1.72)	3.44 (1.64)	0.34
Fruits and vegetables consumed from home-grown	1.01 (1.44)	1.19 (1.55)	0.80 (1.27)	<0.01
Vegetables	3.13 (1.57)	3.02 (1.61)	3.28 (1.50)	0.09
Vegetables consumed from home-grown	0.93 (1.37)	1.10 (1.46)	0.73 (1.22)	<0.01
Fruits	0.22 (0.52)	0.27 (0.60)	0.16 (0.39)	0.04
Fruits consumed from home-grown	0.27 (0.65)	0.27 (0.78)	0.26 (0.42)	0.28
Household demand indicators				
Affordability	3.65 (0.73)	3.69 (0.70)	3.59 (0.78)	0.19
Availability	3.85 (0.70)	3.83 (0.63)	3.87 (0.78)	0.50
Accessibility	3.83 (0.71)	3.84 (0.71)	3.81 (0.71)	0.70
Proximity—the store/place being near you	2.93 (1.16)	2.81 (1.10)	3.08 (1.22)	0.02
Taste	4.01 (0.49)	3.95 (0.43)	4.10 (0.55)	<0.01
Easiness to prepare/cook	4.15 (0.56)	4.07 (0.55)	4.26 (0.56)	<0.01
Knowledge to prepare FV	4.05 (0.63)	3.94 (0.59)	4.19 (0.65)	<0.01
Food safety of FV	3.91 (0.58)	3.77 (0.54)	4.11 (0.57)	<0.01
Household health literacy				
Composite score from the principal component analysis	9.75 (1.42)			
Area of irrigated land (hectares)	2.31 (2.23)	2.19 (2.02)	2.47 (2.50)	0.21
The highest level of education completed by the head of household (Rank from 1 to 8)	3.46 (1.56)	3.43 (1.52)	3.50 (1.61)	0.66
Primary language spoken (%)				<0.01
Odia	44.7	52.05	34.46	
Sambalpur	18.1	19.26	16.38	
Adivashi	29.7	19.67	43.5	
Other	7.6	9.02	5.65	
Social group (%)				0.05
General	23.8	27.46	18.64	
Scheduled caste (SC)	6.7	7.79	5.08	
Scheduled tribe (ST)	47.3	42.21	54.24	
Other backward caste (OBC)	22.3	22.54	22.03	
Household food insecurity (%)				0.21
No	89.9	91.8	88.14	
Yes	10.1	8.2	11.86	

* *The p-value of statistical comparison between low- and high-health literacy groups*.

The multivariable linear regression results for FVC, VC, and FC are shown in Table 2. The study found that people with a high level of home-grown fruits and vegetables consumption (HgFVC) would associate with more FVC (β = 0.65, SE = 0.10, *p* = 0.008), but the household health literacy was not associated with FVC (β = 0.12, SE = 0.07, *p* = 0.21) after adjusting for control variables. The results also presented the similar findings for VC where VC positively associated with home-grown VC (β = 0.57, SE = 0.11, *p* = 0.02), but not with health literacy (β = 0.15, SE = 0.07, *p* = 0.11). Meanwhile, among the demand indicators, both availability (β = 0.29, SE = 0.06, *p* = 0.02) and accessibility (β = 0.06, SE = 0.01, *p* = 0.02) were both positively associated with VC. As for FC, individuals who consumed more home-grown fruits (β = 0.58, SE = 0.05, *p* < 0.01) also reported higher FC. However, there was an interesting result showing that higher health literacy was specifically associated with less FC (β = −0.07, SE = 0.02, *p* = 0.001). When comparing with VC, there were two different demand drivers associated with FC: taste (β = 0.32, SE = 0.11, *p* = 0.005) and easiness to prepare/cook (β = −0.25, SE = 0.09, *p* = 0.006).

**Table 2:** Multivariable model analyses for daily servings per capita of *fruits and vegetables, vegetables only*, and *fruits only*.

	**Fruits and vegetables**						**Vegetables**						**Fruits**					
	**Model 1**			**Model 2**			**Model 1**			**Model 2**			**Model 1**			**Model 2**		
	**β**	**SE**	**P>t**	**β**	**SE**	**P>t**	**β**	**SE**	**P>t**	**β**	**SE**	**P>t**	**β**	**SE**	**P>t**	**β**	**SE**	**P>t**
HgFVC^a^	0.65	0.10	0.008	1.50	0.27	0.01												
HgVC^b^							0.57	0.11	0.02	1.49	0.26	0.01						
HgFC^c^													0.58	0.05	<0.01	−0.89	0.66	0.18
Household demand indicators																		
Affordability	−0.04	0.11	0.70	−0.04	0.09	0.70	−0.10	0.10	0.41	−0.10	0.09	0.38	0.27	0.18	0.14	0.21	0.15	0.17
Availability	0.30	0.07	0.03	0.33	0.08	0.03	0.29	0.06	0.02	0.32	0.07	0.02	0.02	0.12	0.89	0.03	0.10	0.75
Accessibility	0.04	0.03	0.22	0.01	0.04	0.76	0.06	0.01	0.02	0.02	0.03	0.43	−0.02	0.07	0.77	−0.03	0.04	0.39
Proximity	−0.05	0.08	0.58	−0.05	0.08	0.57	−0.10	0.06	0.20	−0.10	0.06	0.21	0.05	0.11	0.62	0.06	0.12	0.64
Taste	0.23	0.12	0.15	0.22	0.14	0.21	0.19	0.13	0.22	0.18	0.14	0.28	0.32	0.11	0.005	0.28	0.10	0.007
Easiness to prepare/cook	0.01	0.14	0.95	−0.01	0.16	0.96	0.04	0.12	0.75	0.02	0.15	0.90	−0.25	0.09	0.006	−0.21	0.04	<0.01
Knowledge to prepare FV	−0.10	0.11	0.44	−0.10	0.11	0.46	−0.04	0.10	0.73	−0.03	0.10	0.76	−0.20	0.14	0.16	−0.19	0.09	0.05
Food safety of FV	−0.23	0.09	0.09	−0.20	0.10	0.14	−0.16	0.07	0.10	−0.12	0.07	0.17	−0.31	0.22	0.16	−0.27	0.18	0.14
Household health literacy																		
The composite score	0.12	0.07	0.21	0.19	0.03	0.01	0.15	0.07	0.11	0.23	0.03	0.004	−0.07	0.02	0.001	−0.15	0.03	<0.01
Interaction = home-grown consumption * health literacy				−0.09	0.02	0.02				−0.09	0.02	0.01				0.17	0.08	0.03
Area of irrigated land	−0.01	0.04	0.75	−0.01	0.04	0.87	−0.02	0.04	0.56	−0.01	0.03	0.74	0.003	0.02	0.87	0.01	0.02	0.48
Education	0.09	0.08	0.36	0.09	0.08	0.34	0.04	0.07	0.59	0.04	0.07	0.59	0.04	0.04	0.30	0.02	0.05	0.70
Primary language spoken																		
Odia	(reference)			(reference)			(reference)			(reference)			(reference)			(reference)		
Sambalpur	0.98	0.14	0.006	1.02	0.13	0.005	0.93	0.19	0.02	0.97	0.18	0.01	0.06	0.08	0.49	0.05	0.06	0.48
Adivashi	−0.09	0.38	0.83	−0.10	0.37	0.81	−0.16	0.49	0.77	−0.17	0.48	0.74	−0.20	0.03	<0.01	−0.18	0.05	0.001
Other	−0.31	0.45	0.54	−0.25	0.40	0.58	−0.10	0.61	0.88	−0.04	0.55	0.95	−1.16	0.13	<0.01	−1.13	0.09	<0.01
Social group																		
General	(reference)			(reference)			(reference)			(reference)			(reference)			(reference)		
Scheduled caste (SC)	0.20	0.36	0.62	0.19	0.32	0.60	0.04	0.37	0.91	0.04	0.34	0.91	−0.13	0.13	0.30	0.03	0.21	0.90
Scheduled tribe (ST)	−0.19	0.40	0.66	−0.18	0.37	0.66	−0.32	0.53	0.59	−0.30	0.50	0.59	0.19	0.13	0.15	0.28	0.11	0.01
Other backward caste (OBC)	−0.23	0.38	0.59	−0.24	0.36	0.56	−0.40	0.19	0.12	−0.38	0.19	0.14	−0.20	0.18	0.27	0.04	0.20	0.86
Household food insecurity																		
No	(reference)			(reference)			(reference)			(reference)			(reference)			(reference)		
Yes	0.48	0.26	0.16	0.46	0.23	0.14	0.51	0.27	0.16	0.50	0.25	0.14	−0.01	0.16	0.96	0.002	0.14	0.99
_cons	0.61	0.95	0.57	−0.21	0.83	0.82	0.20	0.73	0.81	−0.58	0.54	0.36	0.60	0.54	0.26	1.23	0.83	0.14
R-squared	0.39			0.40			0.38			0.39			0.17			0.17		

a *HgFVC, the servings of fruits and vegetables consumed per capita per day from home-grown*.

b *HgVC, the servings of vegetables consumed per capita per day from home-grown*.

c *HgFC, the servings of fruits consumed per capita per day from home-grown*.

As shown in Model 2 on Table 2, the interaction between household health literacy and HGC was significant for each outcome measure: FVC (β = −0.09, SE = 0.02, *p* = 0.02), VC (β = −0.09, SE = 0.02, *p* = 0.01), and FC (β = 0.17, SE = 0.08, *p* = 0.03). According to the subpopulation analyses, HGC was positively associated with overall FVC (β = 0.75, SE = 0.12, *p* = 0.008) and VC (β = 0.69, SE = 0.12, *p* = 0.01) among those who had little health literacy, but not among those who perceived more health literacy (FVC: β = 0.33, SE = 0.11, *p* = 0.06; VC: β = 0.26, SE = 0.09, *p* = 0.06) (Tables 3, 4). In contrast, people with more home-grown FC consume more fruits among both the low- and high-health literacy groups (low: β = 0.56, SE = 0.08, *p* = <0.01; high: β = 0.77, SE = 0.08, *p* = <0.01) (Table 5). Additionally, among the low- and high-health literacy groups, differential demand drivers may have diverse influence on FVC. For instance, more knowledge to prepare FVs could increase VC in the low-health literacy group but reduce VC in the high one. Only easiness to prepare/cook was negatively associated with FC in the low-health literacy group. However, among the high-health literacy group, we found that affordability and easiness to prepare/cook had positive influence, but accessibility and knowledge to prepare FVs had negative influence on FC.

**Table 3:** Subpopulation analyses for the servings of *fruits and vegetables* consumed per capita per day by high-/low-health literacy groups.

	**Low health literacy group**			**High health literacy group**		
	**β**	**SE**	**P>t**	**β**	**SE**	**P>t**
HgFVC^a^	0.75	0.12	0.008	0.33	0.11	0.06
Household demand indicators						
Affordability	−0.11	0.11	0.38	0.12	0.24	0.67
Availability	0.25	0.13	0.15	0.13	0.12	0.36
Accessibility	0.10	0.16	0.58	0.17	0.25	0.54
Proximity—the store/place being near you	−0.01	0.17	0.94	−0.05	0.05	0.36
Taste	0.40	0.17	0.09	0.02	0.17	0.91
Easiness to prepare/cook	−0.15	0.19	0.49	−0.11	0.15	0.50
Knowledge to prepare FV	0.21	0.08	0.08	−0.42	0.08	0.01
Food safety of FV	−0.37	0.26	0.25	0.02	0.11	0.85
Area of irrigated land	−0.01	0.03	0.75	0.01	0.03	0.69
Education	0.05	0.04	0.32	0.09	0.08	0.37
Primary language spoken						
Odia	(reference)			(reference)		
Sambalpur	0.37	0.09	0.03	1.85	0.13	0.001
Adivashi	0.27	0.05	0.01	−0.98	0.25	0.03
Other	−0.44	0.09	0.02	−0.65	0.24	0.07
Social group						
General	(reference)			(reference)		
Scheduled caste (SC)	0.01	0.39	0.99	0.42	0.41	0.39
Scheduled tribe (ST)	−0.29	0.18	0.20	0.41	0.30	0.26
Other backward caste (OBC)	−0.19	0.55	0.76	−0.29	0.11	0.08
Household food insecurity						
No	(reference)			(reference)		
Yes	0.38	0.51	0.51	−0.37	0.19	0.14
_cons	1.06	0.90	0.32	3.54	0.89	0.03
R-squared	0.51			0.44		

a *HgFVC, the servings of fruits and vegetables consumed per capita per day from home-grown*.

**Table 4:** Subpopulation analyses for the servings of *vegetables* consumed per capita per day by high-/low-health literacy groups.

	**Low health literacy group**			**High health literacy group**		
	**β**	**SE**	**P>t**	**β**	**SE**	**P>t**
HgVC^a^	0.69	0.12	0.01	0.26	0.09	0.06
Household demand indicators						
Affordability	−0.15	0.09	0.18	0.06	0.28	0.83
Availability	0.26	0.09	0.06	0.11	0.11	0.39
Accessibility	0.07	0.17	0.73	0.19	0.26	0.53
Proximity—the store/place being near you	−0.08	0.12	0.57	−0.06	0.04	0.26
Taste	0.38	0.15	0.08	−0.004	0.13	0.98
Easiness to prepare/cook	−0.11	0.21	0.64	−0.21	0.17	0.30
Knowledge to prepare FV	0.24	0.05	0.02	−0.33	0.10	0.05
Food safety of FV	−0.23	0.20	0.34	0.09	0.11	0.50
Area of irrigated land	−0.04	0.04	0.38	0.03	0.02	0.19
Education	0.01	0.03	0.83	0.04	0.06	0.57
Primary language spoken						
Odia	(reference)			(reference)		
Sambalpur	0.45	0.04	0.002	1.61	0.10	0.001
Adivashi	0.14	0.19	0.51	−1.17	0.31	0.03
Other	−0.16	0.18	0.44	−0.71	0.29	0.09
Social group						
General	(reference)			(reference)		
Scheduled caste (SC)	−0.18	0.30	0.60	0.44	0.43	0.38
Scheduled tribe (ST)	−0.41	0.13	0.05	0.51	0.30	0.19
Other backward caste (OBC)	−0.38	0.32	0.31	−0.29	0.14	0.13
Household food insecurity						
No	(reference)			(reference)		
Yes	0.45	0.52	0.45	−0.26	0.17	0.22
_cons	0.84	1.06	0.48	3.75	0.99	0.03
R-squared	0.51			0.42		

a *HgVC, the servings of vegetables consumed per capita per day from home-grown*.

**Table 5:** Subpopulation analyses for the servings of *fruits* consumed per capita per day by high-/low-health literacy groups.

	**Low health literacy group**			**High health literacy group**		
	**β**	**SE**	**P>t**	**β**	**SE**	**P>t**
HgFC^a^	0.56	0.08	< 0.01	0.77	0.08	<0.01
Household demand indicators						
Affordability	0.22	0.23	0.33	0.10	0.02	<0.01
Availability	−0.06	0.19	0.76	0.08	0.12	0.51
Accessibility	0.10	0.18	0.56	−0.31	0.10	0.002
Proximity—the store/place being near you	0.07	0.16	0.68	−0.10	0.11	0.35
Taste	0.31	0.36	0.38	0.17	0.26	0.51
Easiness to prepare/cook	−0.28	0.07	<0.01	0.22	0.09	0.02
Knowledge to prepare FV	−0.14	0.15	0.35	−0.59	0.22	0.007
Food safety of FV	−0.44	0.20	0.03	−0.26	0.27	0.35
Area of irrigated land	0.03	0.05	0.60	−0.01	0.05	0.88
Education	0.01	0.09	0.87	0.03	0.02	0.05
Primary language spoken						
Odia	(reference)			(reference)		
Sambalpur	−0.11	0.20	0.58	0.08	0.19	0.67
Adivashi	−0.09	0.17	0.59	−0.02	0.20	0.93
Other	−0.87	0.75	0.24	.	.	.
Social group						
General	(reference)			(reference)		
Scheduled caste (SC)	−0.09	0.38	0.81	−0.07	0.51	0.90
Scheduled tribe (ST)	0.02	0.28	0.95	−0.20	0.25	0.42
Other backward caste (OBC)	−0.22	0.25	0.38	−0.09	0.04	0.009
Household food insecurity						
No	(reference)			(reference)		
Yes	−0.17	0.05	0.002	−0.61	0.10	<0.01
_cons	0.42	1.31	0.75	2.06	0.58	<0.01
R-squared	0.19			0.14		

a *HgFC, the servings of vegetables consumed per capita per day from home-grown*.

## Discussion

This research examined the single and combined contributions of home-grown consumption (HGC) and FVC health literacy in rural India. The evidence from the current study indicates that higher HGC is associated with higher consumption of fruits and vegetables, which confirms previous evidence on the efficacy of agricultural approaches as a way of increasing FVC by promoting HGC (17, 19, 21, 22). Previous research suggested that home-grown agriculture can influence food choices (59), as well as dietary diversity (60), and that it could be one aspect of agricultural strategies to combat low FVC. The mechanism through which this pathway operates is that home-grown production is expected to provide a direct resource of food to be eaten by a household, especially in the context where food marketing cannot function properly (60).

Evidence has shown that homestead farming contributes significantly to nutrition outcomes in developing countries (7, 61). For example, one recent review demonstrates an association between agricultural interventions and nutritional outcomes in South Asia and indicated that homestead gardens could play a crucial role in increasing the consumption of FVs (22). In addition, the results from the study in rural Andhra Pradesh, India, showed that after introducing homestead gardens, households increased the frequency of green leafy vegetable consumption (62). Encouraging home-grown agriculture may not only increase access to fresh FVs for producers and their families but also provide financial savings of purchasing FVs (63), which are part of hypothesized factors contributing to access and affordability for FVs (64, 65). India is one of the largest global producers of FVs, but the majority of production is exported (66). However, food intake of agricultural households still largely depends on farmers' own production in India (22, 67), and evidence has also shown that home-grown agriculture may result in an increased intake of FVs (68). Existing agricultural policy in India mainly focuses on growing government support for increasing FV production, infrastructure development, and agricultural marketing (66). Yet, our results also point to the need for agricultural policies and programs that promote home-grown agriculture as a means to improve FVC.

Previous studies have treated fruits and vegetables in a similar manner when exploring the drivers of FVC. However, recent studies argue that fruits should be considered separately from vegetables given that drivers could be different, and separate strategies may be needed (69–71). Our study has shown that there were differential drivers for fruit and vegetable consumption, which is consistent with previous findings (45, 69, 71–74). In this study, people's perceptions of the availability and accessibility of fruits and vegetables were associated with vegetable, but not fruit, consumption. In India, there are few local markets in rural areas and, thus, more restrictions in the variety of FVs available for purchase (53, 54). Since vegetables are generally less expensive and affordable (75, 76), availability and accessibility may play even more essential roles in encouraging vegetable consumption in India. Fruit consumption was positively associated with taste, but negatively associated with easiness to prepare/cook and health literacy. In eastern cultures, such as India, people believe that foods that are pleasant and fresh in taste could promote health, physical strength, and mental abilities (77, 78), and this belief may result in increasing consumption of food with good taste, such as fruits. There was an interesting finding on the negative association between easiness to prepare/cook and FC, which was inconsistent with previous research (70) and requires further study. Generally, people would increase their fruit consumption once the health benefits of fruits are known (31, 72). Perhaps, farmers who know this fact may choose to sell the majority of the fruits they grow due to their high agricultural revenues (79).

Meanwhile, our findings additionally showed that health literacy could modify the influence of HGC on FVC. Specifically, after stratifying the data into low- and high-health literacy groups, the positive influence of HGC on FVC was demonstrated only in the low-health literacy group. Previous evidence suggests that health literacy is a prominent predictor of FVC (30, 35), and for certain nutritional outcomes, nutritional literacy is of paramount importance, while agricultural practices might be less influential (21). Therefore, the potential explanation for our finding is that people with high-health literacy not only have a higher level of their FVC but also would be more aware of whether they could get the sufficient FVC through multiple sources besides home-grown produce (31, 71). Based on the descriptive statistics in our study (Table 1), people in the high-health literacy group did have higher consumption of fruits and vegetables compared with those in the low group. Yet, the intake level of HgFVC is lower in the high-health literacy group than in the low one. It may be argued that people who have sufficient health literacy on FVs find diverse food sources to consume more fruits and vegetables, instead of limiting themselves to access home-grown FVs, and therefore, HGC might not be directly associated with FVC, whereas, for people who have lower levels of health literacy related to fruits and vegetables, may just consume what they produce. Future research is needed to further confirm this argument.

Meanwhile, the findings also suggest that more vegetable consumption from home grown could increase overall vegetable consumption only among people with low-health literacy. However, the positive impact of home-gown fruit consumption on overall fruit consumption was the same in both low- and high-health literacy groups. Additionally, subpopulation analyses also reveal that differential drivers could be found based on the level of health literacy. These results suggest that when designing an intervention for improving FVC, it would be important to consider differential levels of health literacy among people in order to apply the most appropriate behavioral interventions. For example, knowledge to prepare FVs could have reverse influence on vegetable consumption among low- and high-health literacy groups, and the influence of affordability, accessibility, or knowledge to prepare FVs could be found only in the high-health literacy groups. Different demand drivers in low- and high-health literacy groups may cause different consumption rates of fruits and vegetables among the two groups, as also evidenced by previous studies demonstrating that the determinants of fruit consumption differ from vegetable consumption (45, 69, 71–74). For vegetables, people among the high-health literacy group may try to find the various types of vegetables that meet their demand, and the food source would not be limited from their own agricultural produce. However, fruits are generally more expensive than vegetables and other common foods (75, 80). Therefore, people among both low- or high-health literacy groups may only consume fruits from their homestead as a more affordable approach.

This study has several limitations. First, the results may not be generalizable to other rural settings since the data were collected in only four districts of rural Odisha. Nevertheless, our main findings of differential drivers and consumption motives that differ by fruit and vegetable consumption literacy level have added further knowledge to fruit and vegetable research. Second, measures of fruit and vegetable consumption, as well as health literacy, are partial. FVC is the most obvious one because no instrument has perfectly captured consumption data—and there are often biases when using self-reported measures. Meanwhile, the measure for health literacy had a similar measurement issue. There are various aspects for health literacy, but the focus of the present study was on perceived health benefit on vegetables due to the limitation on the questionnaire. Third, the data in this study were cross-sectional, and causality should not be directly inferred from the findings in this study.

## Conclusion

In India, low consumption of fruits and vegetables undoubtedly constitutes one of the country's critical nutritional problems. Although there is an important role for the agricultural and educational sectors in improving FVC, less is known about their direct and indirect relationships to farmers' decisions about fruit and vegetable consumption. This study filled in the gap of the empirical evidence on the potential contribution of agricultural approaches on FVC, as well as the connection between agricultural and knowledge-based approaches. Results indicated that higher home-grown consumption was associated with improving FVC, with one implication being the importance of agricultural programs as representing viable entry points for increasing FVC. Evidence on the contingent relationship between agricultural and knowledge-based programs highlights the multifaceted approaches needed to promote FVC. Therefore, for countries like India, the policy focusing on behavior or educational approach to increasing FVC was not enough, and the effective policy development need to additionally incorporate agriculture aspect, such as home gardening, to construct stronger synergies to act on nutrition-sensitivity agriculture intervention. In addition, further research on elaborating a clear direction of impact pathways, theories of change, or cost effectiveness of nutrition-sensitivity agriculture intervention is needed in order to translate and provide solid evidence to policy makers.

## Data Availability Statement

The raw data supporting the conclusions of this article will be made available by the authors, without undue reservation.

## Ethics Statement

The studies involving human participants were reviewed and approved by Research Ethics Board of McGill University. The patients/participants provided their written informed consent to participate in this study.

## Author Contributions

Y-HW was a contributor to the study conception, design, data analysis, and wrote the manuscript. CM contributed toward manuscript preparation and revisions. SM and LD contributed toward study conception, design, and data collection. All authors critically reviewed and approved the final manuscript for submission.

## Conflict of Interest

The authors declare that the research was conducted in the absence of any commercial or financial relationships that could be construed as a potential conflict of interest.

## Abbreviations

FC: fruit consumption
FVs: fruits and vegetables
FVC: fruit and vegetable consumption
HGC: home-grown consumption
HgFVC: home-grown fruit and vegetable consumption
NSSO: National Sample Survey Office
VC: vegetable consumption.
